# Cognitive and Physiologic Impacts of the Infraslow Oscillation

**DOI:** 10.3389/fnsys.2018.00044

**Published:** 2018-10-16

**Authors:** Brendon O. Watson

**Affiliations:** Department of Psychiatry, University of Michigan, Ann Arbor, MI, United States

**Keywords:** infraslow, oscillations, resting state network, glia, thalamus, cortex, cognition

## Abstract

Brain states are traditionally recognized via sleep-wake cycles, but modern neuroscience is beginning to identify many sub-states within these larger arousal types. Multiple lines of converging evidence now point to the infraslow oscillation (ISO) as a mediator of brain sub-states, with impacts ranging from the creation of resting state networks (RSNs) in awake subjects to interruptions in neural activity during sleep. This review will explore first the basic characteristics of the ISO in human subjects before reviewing findings in sleep and in animals. Networks of consistently correlated brain regions known as RSNs seen in human functional neuroimaging studies oscillate together at infraslow frequencies. The infraslow rhythm subdivides nonREM in a manner that may correlate with plasticity. The mechanism of this oscillation may be found in the thalamus and may ultimately come from glial cells. Finally, I review the functional impacts of ISOs on brain phenomena ranging from higher frequency oscillations, to brain networks, to information representation and cognitive performance. ISOs represent a relatively understudied phenomenon with wide effects on the brain and behavior.

There is significant evidence that as the brain cycles through states, varying computational functions are affected. The classical brain states of wake, sleep and nonREM sleep have been analyzed in great detail. On the other hand much faster oscillations in frequencies ranging from 1 Hz to 150 Hz typically garner attention by electrophysiologists. Here, I will discuss work on the infraslow oscillation (ISO) an approximately 0.02 Hz (1/min) oscillation that lies in a temporal domain between typical brain sleep/wake state shifts and faster oscillations. The ISO interacts with a variety of neural functions to affect both sleep dynamics and cognition during wake states.

## The Infraslow Oscillation and Brain-Wide Network States

**Figure 1:**
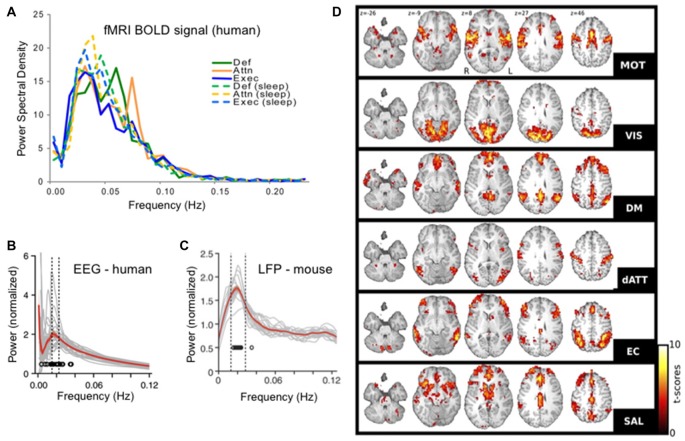
Infraslow brain oscillations in the 0.01–0.06 Hz range and resting state networks (RSNs). **(A)** Fourier spectral analyses of each of six resting state sub-networks in functional magnetic resonance imaging (fMRI) blood oxygenation level dependent (BOLD) signals showing peaks in the 0.01–0.06 Hz range. **(B)** Human electroencephalogram (EEG) and **(C)**. Mouse EEG show modulation of the sigma band (10–15 Hz) power with modulation cycles peaking in the 0.02 Hz range, similar to the BOLD signal. **(D)** Six RSNs found in awake human patients. RSN t-score values represent degrees of correlation between fMRI voxels at the 0.01–0.06 Hz range of the BOLD signal in each voxel. RSNs were determined in a data-driven manner using independent components analysis (ICA). Each RSN is given its own name in the literature and is labeled here accordingly: motor network (MOT), visual network (VIS), default mode network (DM or DMN), dorsal attentional network (dATT), executive control (EC) and salience (SAL). Panel **(A)** reproduced with permission from Larson-Prior et al. (2009). Panels **(B,C)** reproduced with permission from Lecci et al. (2017). Panel **(D)** reproduced with permission from Boly et al. (2012).

The ISO, defined as a brain electrical rhythm with maximal spectral power in the frequencies from 0.01 Hz to 0.1 Hz was first described using depth electrodes in unanesthetized rabbits in 1957 (Aladjalova, 1957). But starting in the early 2000s this rhythm began to receive increased attention, as the oscillation underlying resting state networks (RSNs) in awake human subjects (Fox and Raichle, 2007; Figure 1). RSNs were first discovered using temporal correlations in the blood oxygenation level signals between distant brain regions in functional magnetic resonance imaging (fMRI) datasets. These signals are sampled with a temporal resolution that is relatively low compared to electrophysiologic recordings (often 1 Hz sampling or slower) and by dint of this temporal resolution, any correlations observed were necessarily at frequencies below the usual “floor” of the most commonly studied electrophysiologic rhythms at 0.5 Hz or so. RSNs are therefore networks of co-varying brain regions oscillating between 0.01 and 0.1 Hz, with a peak at 0.02–0.03 Hz (Biswal et al., 1995; De Luca et al., 2006). While RSNs are reviewed more completely elsewhere (Fox and Raichle, 2007) some salient features are worth describing here. First there are many RSNs detectable in a given brain, with many studies finding approximately six distinct significant networks. Each corresponds to different co-varying neuroanatomical regions and some of these networks tend to cluster into regions previously identified by neuroscientists as functionally related (for instance a visual network dominated by posterior regions). On the other hand, some of these infraslow-range oscillating families of regions represent less traditionally predictable functional groups, such as the salience network and dorsal attentional networks composed of regions outside the more studied motor and sensory regions of the brain, thereby revealing and defining basic neuroanatomical functional relationships (Damoiseaux et al., 2006; Boly et al., 2012). Indeed, the notion of RSNs instructing the field of neuroscience regarding brain functional organization is reinforced by the fact that many researchers use unbiased data driven approaches, such as independent component analysis (ICA) to determine these networks—lending credence to their identity as brain-defined families of regions.

After the establishment of RSNs, more recent work has come full circle to link RSN patterns in fMRI to ISO-frequency voltage changes in the electroencephalographic (EEG) signal (Grooms et al., 2017). Yet finer scale analysis showed that ICA-determined fMRI RSNs overlap with ICA-determined EEG networks, both in space and time (Hiltunen et al., 2014).

While ISOs and RSNs were initially described in awake subjects they have also been found during sleep. First, as in wake, 0.02 Hz is a frequency band with peak power in the fMRI temporal signal during sleep (Horovitz et al., 2008; Larson-Prior et al., 2009). It seems that RSNs have both similarities and differences across brain states, with some reports emphasizing differences across electrophysiologically or behaviorally defined brain states (Calhoun et al., 2014; Allen et al., 2018) and others finding gross-scale consistency across long-term epochs of wake and nonREM sleep (Boly et al., 2012). Thus, while fine-scale aspects may differ across states, ISOs and RSNs may have grossly similar structures regardless of underlying milieu.

## Diverse Measurement Tools

As already discussed, the infraslow rhythm has been measured with both electrical and neuroimaging modalities. It also can be seen across sleep, wake and anesthetized states and in both neurons and glia. This on the one hand supports the notion that it is a pervasive and potentially important rhythm, but on the other hand brings up the technical caveat that these signals are not identical, and one should be careful in comparing them.

One can break the recording methodologies referenced here into categories including Blood Oxygenation Level-Dependent (BOLD) signal based, electrical measures of direct infraslow voltage oscillations, measures in modulation of power in voltage oscillatory frequency bands, intracellular measures and calcium imaging measures. Not all of these have been directly correlated head-to-head in a pairwise manner. Furthermore, when they have been compared the correlations are not perfect. In particular temporal correlations in infraslow bands between fMRI and EEG range up to approximate *r* values of 0.4 (Thompson et al., 2014) but were often substantially lower with correlation coefficients around 0.05 in some analyses (Hiltunen et al., 2014).

Much of this may stem from the fact that each of these signals, including BOLD measures or electrical recordings are not singularly dictated by infraslow rhythms but rather each incorporate infraslow rhythms with various other brain-related activity. Those non-infraslow factors are often not correlated and likely carry important information regarding other domains. The fact that varying signals not only have similar frequency band activities but are correlated in time to some extent is indicative that the infraslow rhythm is pervasive.

An additional technical point of note as we proceed: many animal studies are performed under anesthesia and the brain is in a fundamentally different state than when naturally awake or asleep. The infraslow rhythm is able to modulate both the 0.5–4 hz range rhythm of anesthesia, as well as the higher frequency activity of waking states—again indicating a pervasive role for this oscillation. But, it is worth noting the underlying difference in brain milieu existing in the works cited here and I will try to point out brain states whenever possible.

## The Infraslow Oscillation During Human Sleep

**Figure 2:**
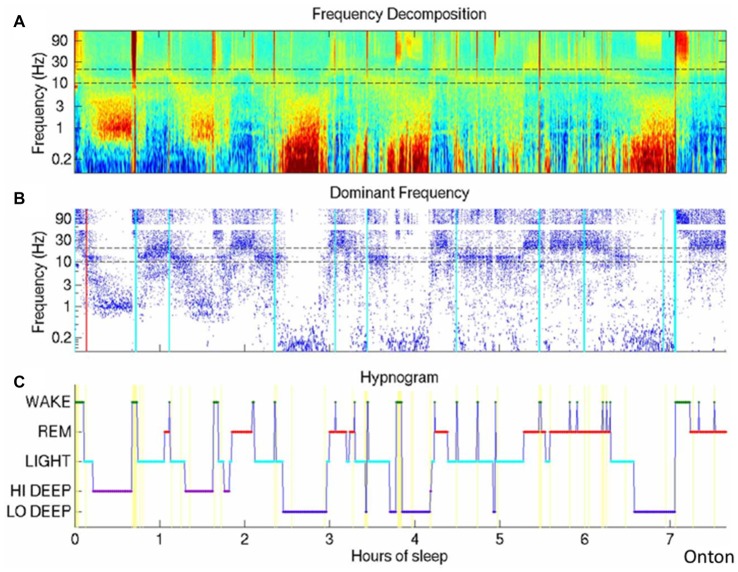
The Infraslow oscillation (ISO) during sleep. **(A)** Wavelet spectrogram of healthy human EEG signal during a sleep session. Horizontally displayed is time, vertically arranged are frequencies and color represent the power in each frequency band at each time point. Infraslow-range (below 0.1 Hz) power is elevated in approximately three epochs around hours 2.5, 4 and 6.5. **(B)** Dominant frequencies for each time point are indicated as blue dots, easily highlighting the periods of highest power in the infraslow frequency. **(C)** Hypnogram demonstrating the traditional WAKE, REM and varying levels of nonREM. The authors here propose a deeper level of deep sleep, which they call LO DEEP, that occurs specifically when infraslow rhythms dominate the power spectrum (all panels reproduced with permission from Onton et al., 2016).

Frank voltage oscillations in the range of 0.01–0.1 Hz are often described with a peak near 0.02 Hz, just as in the waking state. They have been found in epilepsy patients both using scalp EEG and using intracranial electrodes (Achermann et al., 1993; Vanhatalo et al., 2004; Rodin et al., 2014). These oscillations are irregular but clear and more recent spectrographic analyses (Onton et al., 2016) have replicated the finding of low frequency oscillations during human sleep, using wavelet spectrographic analysis. In fact, this wavelet spectrographic approach allows one to clearly visualize the inconsistency of this oscillation from one sleep cycle to the next and the authors suggest a secondary deeper level of deep slow wave sleep wherein the ISO is present (Figure 2). These authors further show that electrodermal activity is also increased only during deep slow wave sleep with high infraslow power.

In addition, the ISO also modulates the power of faster oscillations such that power is high in these other frequency bands during certain phases of the infraslow cycle and low during other phases—an example of cross frequency coupling (Buzsáki and Wang, 2012). Perhaps most clearly and consistently shown to be modulated by the ISO are the delta (0.5–4 Hz) and sigma (10–20 Hz) bands (Vanhatalo et al., 2004; Lecci et al., 2017). Interestingly these two bands relate strongly to two major components of nonREM sleep, slow oscillations in the delta range and spindle oscillations in the sigma range, implying that the major nonREM rhythm generators, and presumably their functions, are modulated by the infraslow generator. Theta band (5–10 Hz; Novak et al., 1992; Vanhatalo et al., 2004), alpha band (8–12 Hz; Novak et al., 1992), gamma band (30–100 Hz; Nir et al., 2008) and multi- and single unit firing (Moiseeva and Aleksanian, 1986; Nir et al., 2008) are also significantly modulated by the ISO—again carrying with them much presumed neural functionality. In addition, this infraslow modulation of other bands may be stronger in sleep than during waking states, at least as measured by the degree to which the ISO couples across large swaths of EEG sites (Liu et al., 2010).

This cross-frequency coupling then actually links the ISO EEG evidence with a phenomenon called the cyclic alternating pattern (CAP) wherein delta, theta and sigma power are modulated irregularly over normal healthy sleep over tens of seconds, falling into the infraslow range (Terzano et al., 2002). The interruptions, or times of low power between high-power periods have been named microarousals (MAs) and were noted to be frequently preceded by K-complexes and spindles (Halász, 1998). They also may be modulated over the course of the sleep cycle (Halász et al., 2004). In fact CAP modulation of these frequency bands is seen most prominently prior to REM sleep (Terzano et al., 1989, 2002) and may link with the notion of “intermediate sleep” wherein spindling is increased just before REM (Gottesmann et al., 1984). Further insight regarding this from animal work will be summarized below. Additionally during CAP episodes heart rate, heart rate variability, respiratory rate and other autonomic markers are all modulated (Parrino et al., 2012). This last observation meshes with the electrodermal findings during deep slow wave sleep with infraslow activity (Onton et al., 2016) and begs the question of whether CAP also varies from one sleep cycle to the next.

The hypothesis that the interruptions in the power of 0.5–25 Hz oscillations by MAs is an arousal-like phenomenon was initiated by Schieber et al. (1971) and later modified and extended by Halász et al. (2004). This approach has led to detection of MA events at the 0.24–0.57 Hz rate—a frequency resembling closely the ISO and CAP events (Novak et al., 1992; Achermann and Borbély, 1997). The physiologic findings during these MA events were variable and this was allowable under the notion that arousal systems in the cortex, subcortical systems and autonomic centers could be engaged variably from event to event and only the strongest arousals included all systems. Specifically, the authors used criteria including reduction in power of low frequency bands in the EEG, awakening, increased muscle tone, change in autonomic state (increased blood pressure, increased heart rate variability). Under this arousal-based conceptualization, MAs are a natural part of sleep which are both arousal-like but usually are not correlated with consciousness. While increased MAs are found in those with insomnia, some MAs are found in healthy natural sleeping subjects (Halász et al., 2004). Aside from electrographic similarities to the awake state, these MA events are similar to wake in that they can be induced by external sensory stimuli and are frequently in fact preceded by K-complexes. Under the classic definition of K complexes thalamo-cortical electrical events are elicited when noise impinges on the subject, but many MA are preceded by K complexes without an evident sensory input. Both cases may somewhat arouse corticothalamic wakefulness while protecting from full wakefulness (Halász et al., 2004).

## The Infraslow Oscillation During in Animal Studies—Focus on Sleep

Since the initial studies in the cortex of anesthetized rabbits (Aladjalova, 1957), the ISO has also been seen in many non-cortical regions during anesthesia including prominently in the thalamus, as well as in the hippocampus, basal ganglia, thalamus, locus coeruleus, dorsal raphe and olivary nucleus, in many cases under anesthesia (Hughes et al., 2011). A recent study however examined the fluctuations in delta rhythm power and sigma rhythm power both in human EEG and mouse local field potential (LFP, an intracranial analog of EEG) during natural nonREM sleep and found that in both rodents and humans a spectral peak was seen at 0.02 Hz (Lecci et al., 2017; Figure 1). In both species they also showed correlation in the temporal co-modulation of these bands—indicating a single rhythm co-modulating delta-sigma power at once and operating similarly in humans and rodents. On the other hand, the modulation of the delta and sigma bands in nonREM sleep with an approximately 0.02 Hz rhythm has has been at times conceptualized as an interruption in the power of these frequency bands, rather than an oscillation. This characterization may be in part given the non-sinusoidal and irregular nature of the infraslow rhythm wherein the phase of increased delta and sigma is much longer than the low power phase (Watson et al., 2016; Lecci et al., 2017; Miyawaki et al., 2017). Later in this section we will focus on the portion of the cycle between interruptions, but for now we will focus on the low-power interruptions themselves.

Over the decades scientists have given a variety of names to these interruption events in the LFP power spectrum, usually in rats, including “arousal-like periods” (Roldan et al., 1963), “low-amplitude sleep” (Bergmann et al., 1987), “microarousals” (Halász et al., 2004; Watson et al., 2016) or LOW states (Miyawaki et al., 2017), each with slightly varying criteria and interpretation. Similar states of low amplitude during wake were also noted by rat researchers and were given names including “low-amplitude irregular activity” (Pickenhain and Klingberg, 1967) and “Small Irregular Activity” (SIA; Vanderwolf, 1971; Whishaw, 1972). Here I will use the term “interruption” to broadly address all of these brief though inhomogeneous states of decreased spectral power in the 0.5–200 Hz range. It is worth noting a particular inconsistency in the literature relating to “SIA”: it has at times denoted both interruptions in wake and sleep even though SIA was initially described during wake and some work even tried to specify a new term, S-SIA for sleep (Jarosiewicz et al., 2002). The fact that these interruption states occur both in wake and in sleep in the rodent literature is similar to the findings across wake and sleep in humans. The states identified in these studies all share the features of a duration from about 5–40 s, and their power spectrum is rather similar to quiet waking in that they do not demonstrate either the high 0–25 Hz power of nonREM sleep or K-complexes, nor do they demonstrate specific 4–8 Hz theta band power increases seen during active wake or REM sleep. Spectral analyses confirm that rather than specific modulation of each band as described in human studies, these LFP recordings show infraslow interruption of all bands, spanning continuously from 0.5–25 Hz and even up to 50 Hz (Jarosiewicz et al., 2002; Watson et al., 2016; Miyawaki et al., 2017). These descriptions come from recordings in either dorsal hippocampus or neocortex and additionally these events have been described in various species ranging from rodents to monkeys to cats (Halász et al., 2004). Interruptions in REM rhythms also are very well known to occur and have been noted, but are so variable that it is unclear whether or how REM interruptions relate to wake and nonREM interruptions.

**Figure 3:**
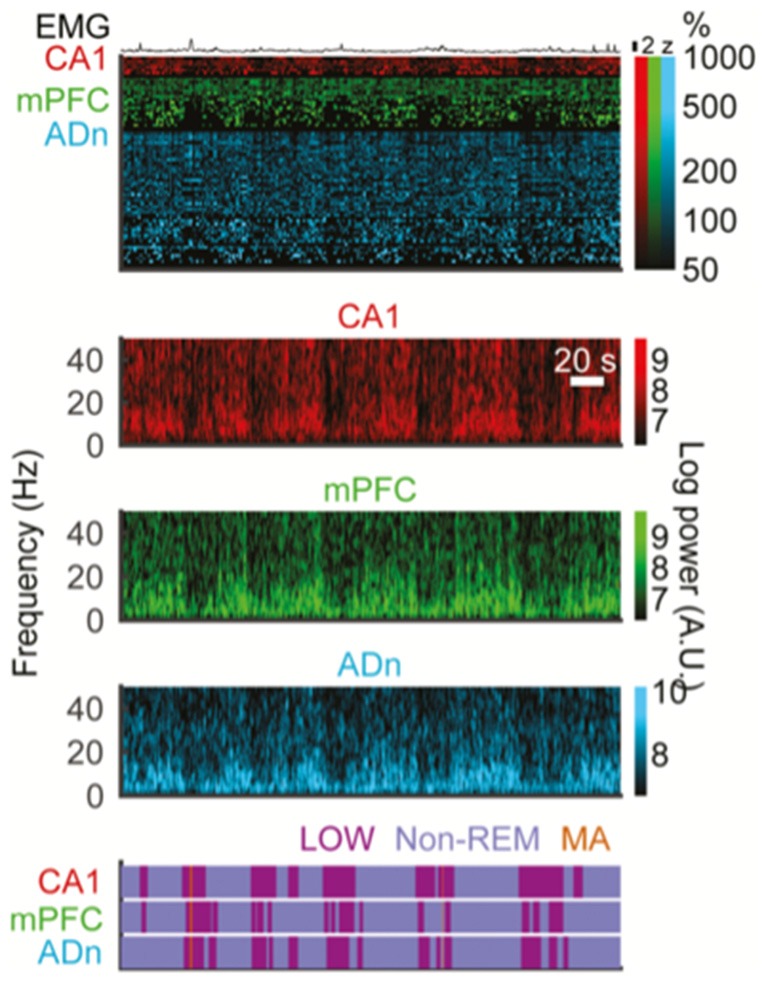
Interruptions in spectral power happen at infraslow incidence rates and simultaneously across forebrain structures. All data here are from multi-electrode recordings in freely behaving mice. Top EMG trace. Top colored block: raster plots of action potentials events from isolated single units in CA1 hippocampus, medial prefrontal cortex (mPFC) and anterior dorsal nucleus of the thalamus (ADn) during simultaneous recordings across all three structures. Lower color blocks are spectrograms showing time-frequency plots of spectral power for local field potential (LFP) recordings in CA1, mPFC and ADn, respectively. Brighter colors denote greater spectral power. Note roughly simultaneous drops in broadband spectral power in all three regions (vertical darkened areas). Bottom purple block: detection of interruption states based on spectral power, performed separately in each region. Again, note overlap of interruption states across these three forebrain regions at once. Note in the top block that spiking patterns are also affected by interruption periods and that EMG elevations tend to occur during interruptions more than outside interruptions (reproduced with permission from Miyawaki et al., 2017).

Recently, Miyawaki et al. (2017) were able to analyze data from many brain regions and using multiple criteria to attempt to synthesize the literature on the various interruption states occurring at infraslow incidence rates (Figure 3). They began by looking for nonREM specific epochs of low power in the 0.625–50 Hz range; they called these “LOW states” and related those to EMG-containing MA events, SIA during wake and sleep, and quiet sleep itself. They found that these states overlapped and perhaps formed a continuum, but again were all linked by having low power in the low frequencies. While many LOW states did not contain EMG activity as in MAs, many MAs and LOW events overlapped, with those overlapping tending to have shorter duration. Furthermore in keeping with the historical literature, these authors defined SIA as low power events during wake, but otherwise were defined similarly to LOW (which were perhaps the same as S-SIA states in prior work (Jarosiewicz et al., 2002)). These two interruption types, essentially only differing in the sleep-wake state in which they are defined show many similarities, though there were statistically significant differences in firing rate, cross-regional coherence and even LFP spectrum. Additionally, they find that LOW events during sleep happen generally but not perfectly synchronously across the hippocampus, frontal cortex, entorhinal cortex, post-subiculum and anterodorsal thalamus (Figure 3). Based on this work, then, as a synthesis of other works, it seems that the formulation put forward by Halasz in 2004 of a continuum of variable states in the human literature may be accurate for rodents: spectral interruption states occur widely across many regions of brain and with some variability in which regions and systems they engage, but fundamentally they represent a continuum of similar events. Similar notions come from recent wake-based rodent work (Hulse et al., 2017).

**Figure 4:**
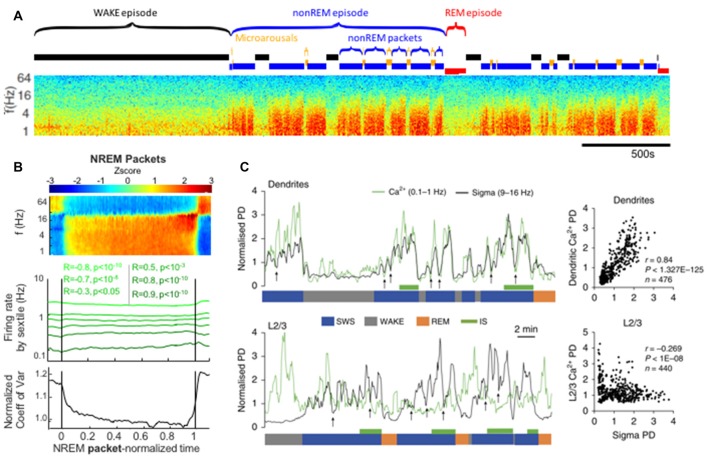
Packets of nonREM activity between interruptions in spectral power correlate with increases in spindle power and dendritic calcium. **(A)** Approximately 1 h time-frequency spectrogram of rat intracortical LFP recording. Labeled above are sleep-epochs. Of note are alternating periods of elevated power in the low frequencies (red blocks in the spectrogram) alternating with interruptions, these are labeled as nonREM packets and Microarousals (MA), respectively and may represent two phases of the infraslow rhythm during nonREM sleep. **(B)** Averaged neural activity during nonREM packets, from all packets across 27 recordings from 11 rats. At top is an average spectrogram showing an increase in sigma-band power as the packet proceeds. Middle shows the firing rates of populations of neurons sorted by their activity prior to the packet, note the convergence of these firing rates towards a middle value by the end of the packet. Bottom panel shows a statistical quantification of this convergence over the packet: the coefficient of variation of the activity rates across the population decreases as the average packet proceeds. **(C)** Simultaneous electrical LFP recordings with calcium imaging. Top: sigma-band power is plotted in black and dendritic calcium is plotted in green. Dendritic calcium is measured after monitoring of the cortical layer 2/3 dendrites of layer 5 cortical neurons expressing GCaMP6s. Note that dendritic calcium and sigma power fluctuate in a correlated manner and do so according to packets (bottom blue SWS label) that are paced by infraslow rhythms. Correlation plot at far right. Below: sigma power vs. calcium in the somatic compartment of layer 5 neurons. These two measures are uncorrelated. Panels **(A,B)** reproduced from Watson et al. (2016). Panel **(C)** reproduced with permission from Seibt et al. (2017).

Looking at the ISO from a different perspective, we can examine the activity of nonREM sleep between the interruptions. In recent rat studies, consistent blocks of 0–25 Hz activity (i.e., between interruptions) were termed “packets” and could terminate in an interruption state, REM sleep or wake (Figure 4; Watson et al., 2016; Seibt et al., 2017). However, within this definition, packets themselves were not uniform states, but rather showed characteristic evolutions in the LFP. Delta and sigma band power both increased as each packet progressed, but this was stronger for the sigma band. There was a specific peak in both sigma-band power and frank spindles detected immediately before the end of each packet, whether that packet was followed by a MA or a REM state. While it has been long noted that spindle power increases before REM and has been named “intermediate sleep” (Gottesmann et al., 1984), the ramp-up of spindle power also prior to MA is an extension of this concept that may point to a more general sleep organizational principle wherein spindle power ramps up in anticipation of any termination of nonREM activity. Furthermore, gamma band power, between 40 Hz and 100 Hz is modulated in counterphase with these low frequency bands, specifically decreasing during packet periods. This likely results from the gamma power drops during each ~1 Hz delta wave during intense slow wave sleep. This orchestration of oscillatory activity both in and outside of the interruption epochs of the ISO can be seen as a faster time-scale sub-rhythm within larger periods of nonREM sleep that organizes nonREM sleep again similar to that described in CAP in humans (Terzano et al., 2002).

In addition to these electrophysiologic animal findings, in the past 10 years it has been reported these ISOs engage RSNs in animals. RSNs have been found in awake monkeys paralleling the human findings (Hutchison et al., 2011), and rodent studies have shown ISOs or RSNs in both anesthetized and wake states (Sforazzini et al., 2014; Stafford et al., 2014; Liang et al., 2015; Hsu et al., 2016). Despite the species difference and the anesthetized states in rodents, both monkey and rodent work show RSNs with similar neuroanatomical distributions to those in humans, based on common brain atlas mapping (Hutchison et al., 2011; Sforazzini et al., 2014; Stafford et al., 2014; Hsu et al., 2016). Additional work shows a linkage between these wide-scale brain activation patterns at infraslow timescales with gamma-band oscillation power (Thompson et al., 2015). One hint that such RSNs, with their variable engagement across the brain, may exist in natural rodent sleep is the variable neuroanatomical engagement of LFP power interruption events shown both in sleep and in wake in rodents (Hulse et al., 2017; Miyawaki et al., 2017). Therefore at this point there is no reason to believe that RSNs are fundamentally different in any mammal than in humans, though certainly more work will be required to fully characterize these infraslow network activations in animals in a manner that allows detailed exploration.

The similarity of both the occurrence and nature of the ISO across wake and sleep states implies a possible common underlying mechanism that acts consistently regardless of larger brain state.

## Mechanisms of the Infraslow Oscillation

The thalamus and glia, and perhaps thalamic glia may be key to the mechanism of the ISO in mammals. Much of this work has been very nicely reviewed already (Hughes et al., 2011), but I will briefly summarize here and will expand based on newer findings.

Thalamic recordings in anesthetized animals and in brain slices show a prominent ISO and these are recognized via paroxysms of action potentials (Destexhe et al., 1998; Lőrincz et al., 2009), LFP fluctuations or intracellular membrane potential oscillations (Lőrincz et al., 2009). While I am not aware of an examination of such thalamic activity in naturally sleeping animals, the fact that sigma-band power and spindles, which are highly thalamus dependent, are modulated in natural sleep at infraslow frequencies is suggestive of thalamic modulation by the ISO in the intact brain (Halász et al., 2004; Watson et al., 2016; Lecci et al., 2017; Seibt et al., 2017). Additionally, the ability to optogenetically induce spindle rhythms in the thalamus of the anesthetized brain was found to be modulated by an underlying infraslow pacer (Barthó et al., 2014), supporting the connection between natural spindles and anesthetized ISOs. Finally, an infraslow modulation of the power of 9–13 Hz band in waking freely moving cats has also been described in the thalamus directly (Lőrincz et al., 2009).

**Figure 5:**
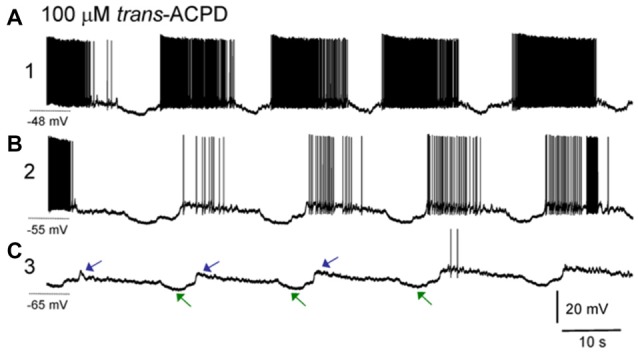
ISOs in thalamic slice recordings. Sharp electrode intracellular recording of a lateral geniculate nucleus (LGN) neuron. **(A)** After application of the group I/II metabotropic glutamate receptor agonist trans-ACPD an infraslow rhythm is induced wherein neurons fire paroxysms of action potentials, with slowly decrementing rates, with punctuated hyperpolarizing events. Note the asymmetric phase of this oscillation with a long firing phase and shorter interruption phase, similar to the asymmetric oscillation seen in nonREM sleep recordings. Holding potential during this phase of recording was −48 mV. **(B)** Same neuron after direct current injection to adjust resting potential to −55 mV. **(C)** Same neuron after direct current injection to adjust resting potential to −65 mV. Note that at more hyperpolarized potentials, action potentials are largely absent, but the characteristic hyperpolarizing potentials (green arrows) as well as subsequent depolarizing potentials (blue arrows) composing an infraslow rhythm remain. The cycle time here is approximately 30 s, or 0.03 Hz (reproduced with permission from Lőrincz et al., 2009).

In the brain slice preparation, an ISO appears after bath application of muscarinic agonists and/or metabotropic glutamate agonists. What is manifested by this rhythm are strong and defined hyperpolarizing events in intracellularly recorded neurons, but which are not fully synchronous across those neurons, having times delays of many seconds between cells. Interestingly, the hyperpolarizing intracellular events are correlated with downgoing, not upgoing LFP waveforms, implying that this LFP may not be generated by summated activity of the thalamic neurons. These hyperpolarizations correlating with incoming LFP currents together with the long propagation delays of seconds (rather than typical synaptic millisecond timescales) between neurons suggests that the thalamic neurons themselves may not drive this oscillation (Lőrincz et al., 2009; Figure 5).

Ultimately these hyperpolarizations may be glially-mediated. First, the hyperpolarizing rhythm is blocked by bath application of barium, a blocker of inwardly-rectifying K^+^ channels. Subtypes of these channels may be activated by adenosine A1 receptors and an A1 receptor blocker, DPCDX, also blocks the hyperpolarizing rhythmic events (Lőrincz et al., 2009). Relatedly glia in the thalamus may activate A1 receptors via release of adenosine triphosphate (ATP) after glial calcium events. These glial calcium events have been measured to occur at infraslow frequencies of approximately 0.01 Hz (Parri et al., 2001) in slices. Recent *in vivo* measurements has shown that glial waves in the hippocampus occur on a minutes (infraslow) timescale, that they last for tens of seconds, similar to infraslow interruption events (LOW or MA or SIA) and that when the waves occur, LFP power in the hippocampus is globally reduced, implying that glial waves may underlie infraslow-rate interruptions in neural LFP (Kuga et al., 2011). Additionally cortical glial activity increases specifically at times of LFP spectral power interruptions in natural sleep (Poskanzer and Yuste, 2016). Finally, activation of astrocytes using archaerhodopsin statistically significantly induced shifts in cortical LFP spectra, usually increasing delta-band oscillations, but sometimes reducing delta oscillatory power (Poskanzer and Yuste, 2016). It is possible that the variability in state-shifts from these stimulation experiments was due to the phase of the ISO in which the stimulus was given. These findings are perhaps similar to optogenetic results, wherein spindles could only be induced at certain phases of an underlying ISO (Barthó et al., 2014). These findings may both indicate that the ISO has a controlling role in brain dynamics.

On the other hand one study found that LOW, and possibly MA events were found to occur at lower rates when “sleep pressure” was higher—sleep pressure being a term to connote the drive to sleep and which is measured by delta oscillatory power and which should decrease as sleep proceeds (Miyawaki et al., 2017). In this study sleep pressure was measured essentially as increased oscillatory power, and via sleep cycle counts, and via both metrics higher sleep pressure correlated with fewer interruption events. It has not been explored whether this could be a masking effect wherein greater delta oscillatory drive prevents that oscillation from being interrupted as easily, despite a normal ISO, or whether a separate mechanism actually alters the ISO itself. Either way this does suggest some interplay between the ISO and brain state more generally and stands in at least some contrast to the concept of similar ISOs across sleep and wake.

While much work remains to be done, it seems reasonable to hypothesize that glial networks may underlie the ISO. Stimulation of glia leads to LFP spectral shifts, and naturally occurring glial events coincide with LFP power interruptions. Even outside the context of the intact brain these glial events occur at infraslow frequencies and may induce correlated thalamic hyperpolarizing events of the same incidence frequency. These events also may occur in hippocampus and cortex, but the thalamus shows an independent and strong infraslow rhythm and furthermore may underlie the brainwide patterns seen in RSNs via coordinated nuclei within thalamus (Halassa et al., 2014). Ultimately it may be the effect of rhythmic glial events upon thalamic neurons that affects both a widespread interruption in LFP activity and the variety of RSNs.

## Neural and Computational Effects of Infraslow Oscillations

The ISO has an impact on both neuronal activity and behavior. In fact it is frequently measured and identified by its impact on faster time-scale brain rhythms, including sigma band, delta band and gamma band rhythms (Vanhatalo et al., 2004; Lecci et al., 2017). It is not yet clear how these rhythms are impacted by the ISO, but many of these rhythms are considered stable network regimes under certain physiologic and network parameter states (Sanchez-Vives and Mattia, 2014; Jercog et al., 2017; Levenstein et al., 2018) and so it may be that the phases of the ISO correspond with alterations in these background conditions. It is clear however that these higher-frequency rhythms in turn impact both spike rates and spike timing on a broad population scale (Jarosiewicz et al., 2002; Buzsáki, 2010; Miyawaki and Diba, 2016; Hulse et al., 2017; Watson et al., 2018). The infraslow cycling within nonREM sleep may actually also play a particular role in the homeostatic effects of sleep, with one study finding that alternating nonREM packets and MAs may cooperate to homogenize the firing rates of populations of excitatory neurons over sleep (Watson et al., 2016). In fact synaptic potentials and population responses in the dentate gyrus after stimulation of the perforant pathway are also modulated according to the phase of the ISO (Dash et al., 2018), though further work has not yet been performed to further elucidate the import of this modulation.

In addition to these population-wide effects there are indications that the ISO may affect both coding and learning. First, it is well known that the delta and spindle oscillations modulated by the infraslow play a role in learning and reconsolidation during sleep. Delta oscillations have recently been causally shown to play a role in refining network representations in a manner that aides in cortical learning (Gulati et al., 2017). Spindle oscillations, the 10–20 Hz oscillations visible during nonREM in the cortex, hippocampus and thalamus are increased in frequency after learning new information (Johnson et al., 2010). Furthermore and interestingly these events are highly thalamically mediated (Wang et al., 1995; Halassa et al., 2011), possibly connecting with a thalamic mechanism for the ISO. Additionally, as spindle/sigma band power increases within each packet of slow wave sleep, dendritic calcium in superficial cortical layers has been found to increase (Seibt et al., 2017; Figure 4). The fact that spindles are already correlated with learning and that dendritic calcium has been frequently associated with learning (Bittner et al., 2017; Gerstner et al., 2018; Roelfsema and Holtmaat, 2018) firstly implies that perhaps it is via calcium changes that spindles mediate plasticity, but again this is modulated by the ISO dictating sleep structure. Indeed, the sequencing of sleep sub-states is often interpreted as being structured to optimize information and memory processing (Watson and Buzsáki, 2015; Navarro-Lobato and Genzel, 2018) and perhaps the repetitive and specific structuring of spindle bouts prior to infraslow interruptions is important in the structuring of plasticity events relative to other phases of sleep.

Furthermore, during the interruption-phase of the infraslow nonREM fluctuation, there is evidence of activity and information encoding by neuronal populations. First, in the hippocampus a population of neurons fire specifically during nonREM interruption states (Jarosiewicz et al., 2002; Miyawaki et al., 2017) and in fact those neurons carry spatial information regarding the location of the animal when it went to sleep (Jarosiewicz et al., 2002; Jarosiewicz and Skaggs, 2004; Kay et al., 2016). Furthermore these neurons may be over-represented in CA2 and those CA2 cells may have the unusual quality of coding for spatial activity specifically during immobile wake epochs (Kay et al., 2016). One idea is that while most neurons are tuned to active periods, such as running during wake or ripple-slow wave epochs during sleep, other neurons are responsive to both quiet wake (immobility) and quiet sleep epochs (interruptions in oscillatory activity), periods supplied by the ISO. It may be that the information transmitted during these infraslow-occurring interruption states is unique and important for proper decision-making and context.

In addition to these likely healthy brain processes, the ISO both affects and is affected by pathological conditions. Epileptic events in rodents (Penttonen et al., 1999) and interictal pathological events in epilepsy patients (Vanhatalo et al., 2004) are both phase-modulated by the ISO. Additionally two specific RSNs, the default mode network (DMN, more below) and the central executive network are altered in patients with major depression and those alterations in the DMN are reversed by treatment of depression using transcranial magnetic stimulation (Liston et al., 2014). Also, in patients with attention deficit/hyperactivity disorder (ADHD), spontaneous 0.02–0.2 Hz power is approximately 10% lower than in patients without ADHD (Helps et al., 2010).

RSNs themselves, as networks spanning the brain and engaged specifically by the ISO represent a fascinating mixture of an oscillation with variable anatomical engagement, in a manner that could have functional implications. There is much evidence that the RSNs are based largely on anatomical connectivity across the brain, both in humans (Greicius et al., 2009; Honey et al., 2009) and in rodents (Sforazzini et al., 2014; Hsu et al., 2016), although at least one study found some RSN correlations not predictable by anatomy—implying some alternate mode of coordination (Honey et al., 2009). Regardless of the precise mechanism this intermittent engagement of brainwide sub-networks by this ISO may play an important role in information processing. For instance, it may either reflect the current state of network engagement, or it may help set the background state of the network for future processing—perhaps to prime the brain to be attuned to a certain input stream or to use a particular mode of operation. Probably the most well-known of the RSNs is the DMN, which becomes active in times of introspection, self-generated thought, attentional engagement, as well as during certain electrophysiologic events including sharp wave ripples (Andrews-Hanna et al., 2014). The DMN is an exemplar of the reflection of an infraslow-modulated state which clearly correlates with a particular brain mode of function. The relative engagement of other RSNs also show correlation with what appear to be future behavioral outcomes especially in sensory response tasks. One difficulty in these experiments results from the slow modulation of the BOLD signal by brain activity—meaning that fMRI signals can actually occur after the behavior they temporally precede (Weissman et al., 2006). Given this caveat, it was found that some tasks are correlated with activation of certain RSNs and inactivation of others, including the DMN (Weissman et al., 2006; Boly et al., 2007). Furthermore, the power of the differential RSN engagement in correct vs. incorrect behavioral trials is often sinusoidally modulated at about 0.1–0.02 Hz (Weissman et al., 2006; Sadaghiani et al., 2009).

RSNs may be engaged somewhat differently during sleep than during wake. An EEG study showed first, that ISO more effectively couples oscillatory power fluctuations across the brain in sleep than in wake (Liu et al., 2010). Furthermore, two studies both indicate that anatomical engagement of brain networks by RSNs is roughly grossly analogous between nonREM and wake states, implying that ISOs continue to operate across states (Boly et al., 2012; Mitra et al., 2015). However the DMN appears to be particularly affected by increasing depth of sleep, losing its usual structure (Horovitz et al., 2009; Sämann et al., 2011) and a further study found generalized degradation (though not complete loss) of correlation structure as sleep deepened (Tagliazucchi et al., 2013b). Further analyses of how RSNs generally change during nonREM reveals that there is a shift towards greater within-RSN subnetwork engagement (Boly et al., 2012; Tagliazucchi et al., 2013a). As compared to wake RSNs, nonREM RSNs showed a more modular interaction structure, meaning that compared to wake, the interaction within RSN sub-modules was greater than that across RSNs. RSNs were more modular and internally focused during sleep, but no less active. Indeed nonREM sleep ISO activity, as measured by fMRI is as powerful as the most powerful active waking epochs and is significantly more powerful than quiet waking periods (Horovitz et al., 2008; Larson-Prior et al., 2009). A later study then found that many of the anatomical lead-lag relationships changed or even flipped during the switch from wake to deep nonREM, despite the continued presence of ISO-driven RSNs (Hiltunen et al., 2014). This more complex engagement of RSNs may relate to rodent findings describing more engagement of groups of neurons during nonREM that is more internally generated than during wake (Luczak et al., 2009) and may reflect a different computational process executed by the brain during nonREM.

**Figure 6:**
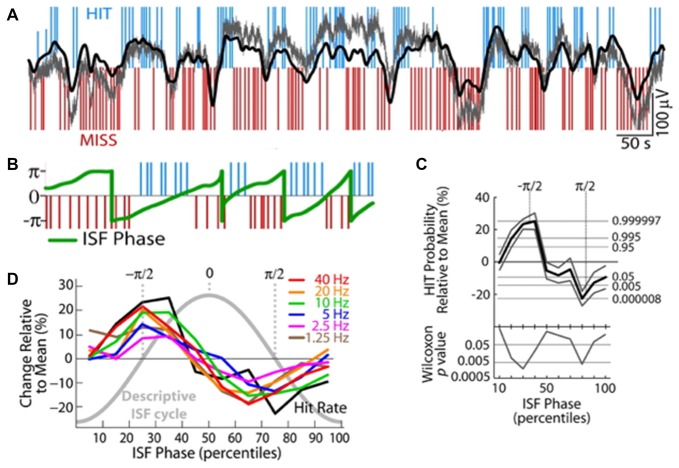
Infraslow fluctuations correlate with fluctuations in human behavioral performance. **(A)** Blue and red lines respectively indicate successes (HITs) and failures (MISSes) by a human subject in detecting sensory stimuli during a sensory task. Overlaid in gray is the integrated power of all frequency components 0–200 Hz of the EEG, and overlaid in black is EEG voltage bandpass filtered at 0.01–0.1 Hz. Elevated or rising phases of the rhythm are more coincident with HITs, low or falling phases are more coincident with MISSes. **(B)** Plot of the phase of the infraslow rhythm (green) superimposed on HITs and MISSes. **(C)** Quantification of relationship between phase of the infraslow rhythm and HITs and MISSes. −π/2 correlates with the highest HIT probability, while π/2 correlates with highest MISS probability. **(D)** HIT Rates (black) are correlated with infraslow phase, as are the powers of various frequency bands (colors; reproduced with permission from Monto et al., 2008).

Waking cognitive processing also interacts with both ISO and the RSNs they induce. In one series of studies, when human subjects were asked to engage in a reaction time task, EEG-measured ISO power decreased approximately by 20% (Helps et al., 2010)—and notably that drop was less in patients with ADHD, settling to a similar eventual task-related power, but having started at a lower power during rest in patients. Furthermore, a Low-resolution brain electromagnetic tomography (LORETA)-based analysis to source-localize EEG signals pointed to specific drops in the ISO power in regions overlapping with the DMN (Broyd et al., 2011). This inactivation of the DMN-related regions is consistent with the concept that DMN is active during non-task periods, but the fact that the ISO itself becomes damped by task engagement is novel. Perhaps complimentarily, the ISO seems to control task performance. Specifically, two studies have found that cognitive task performance correlates with ISO phase. One study found that a modulation in spike rates of locus coeruleus units predicts false alarm rates in monkeys asked to respond properly to rare events, with epochs of increased errors correlating with periods of increased spike generation in the locus coeruleus (Usher et al., 1999). No clear oscillation is demonstrated in this article, but spike rate modulations are shown at infraslow frequencies. A second study, more directly addressing ISOs demonstrated that EEG-based ISO phase predicted “runs” of multiple HITs or MISSes in a sensory detection task in human subjects (Monto et al., 2008; Figure 6). This task was tuned to be detectable at a 50% rate, rendering HITs and MISSes equally likely. The ISO phase also directly correlated with phase of power modulations of all tested frequency bands. It is possible that this infraslow correlate with behavioral output is mediated via DMN engagement, as DMN should usually down-modulate during active tasks, though other mechanistic explanations of course remain.

## Conclusions and Future Directions

The ISO typically peaks in power at 0.02 Hz, is seen in multiple brain regions in mammalian species from mice to humans and occurs across wake and sleep. It organizes neurons, oscillatory patterns, sleep patterns, brainwide networks, is modulated by and modulates both pathological conditions and cognitive performance. Furthermore, the thalamus and in particular a glial effect on the thalamus is a candidate for the generation of these rhythms.

Despite the ubiquity of this rhythm and its broad neural impact, we have only begun to describe how it works and what it does, not to mention why it is present or has the effects it does. Is it “simply” a glial rhythm that neuron-oriented neuroscientists can ignore? Is it an epiphenomenon or did it evolve to serve a purpose? Is that purpose related to neural processing or is it purely metabolic or otherwise? Regardless of origin, are its impacts on neural populations adaptive? Without causal experiments, for instance ablating this rhythm or inducing it, it will be difficult to definitively answer some of these questions. But as of now it has been shown to impact the brain at many levels from spiking to performance, to plasticity windows and disease. It seems we should seriously consider this rhythm and learn from it.

Rodent studies could be instrumental in answering questions about mechanism such that optogenetic or chemogenetic tools can be aimed at the thalamus or other structures to attempt to inactivate the ISO. The additional hypothesis that thalamic subnuclei or the thalamic reticular nucleus coordinate grouped brain structures to create unique RSNs, will require creative experimentation to study but may be addressed with fMRI or electrophysiologically.

We have yet to determine how detailed neural processing is affected by ISOs, either in wake or sleep. In fact, new and interesting directions are opened up especially in the sleep field by the recent findings of dendritic calcium modulation by nonREM packets. Additionally, while there are findings of similarity of engagement of ISO with brain structure and function across states, there are also indications of some degree of modulation by brain state—sleep pressure altering likelihood of events, nonREM altering content of RSN engagement, cognitive state altering engagement. These hints will likely be further explored over time, but they imply a possible dynamic functional impact of ISOs.

What purpose might an oscillation at the timescale of a minute (0.02 Hz) serve? We see it has impacts, but why? Halasz et al proposed that the brain needs rest at times and that perhaps the ISO provides that Halász et al. (2004). Data from rodent researchers implies that perhaps the varying coding during the varying phases of this task may provide the brain with different classes of information, perhaps opening up more broad computational options to brain circuits. Are RSNs the key to understanding the ISO—somehow by engaging these broad-ranging networks, a backbone of basic neural information flow is reinforced for various uses?

The ISO undoubtedly has much more to teach us. It is a broad-ranging and impactful brain rhythm that is likely to receive increased attention in future years.

## Author Contributions

BW conceptualized, researched and composed the document and figures.

## Conflict of Interest Statement

The author declares that the research was conducted in the absence of any commercial or financial relationships that could be construed as a potential conflict of interest.

## Abbreviations

ADHD: Attention Deficit Hyperactivity Disorder
CAP: Cyclic Alternating Pattern—a starting/stopping pattern of power in low frequency oscillation bands
DMN: Default Mode Network—an RSN (below) with increased power during times of non-external attentional focus
EEG: Electroencephalogram
fMRI: Functional magnetic resonance imaging
ICA: Independent Components Analysis
LFP: Local Field Potential—an EEG-related signal measured from inside the brain
LORETA: Low-resolution brain electromagnetic tomography
MA: Microarousal—a brief interruption in nonREM sleep
RSNs: Resting State Networks—groups of brain regions defined by temporal correlation in the infraslow band
SIA: Small Irregular Activity—periods of low amplitude LFP in hippocampus.
